# Healthy lifestyle, DNA methylation age acceleration, and incident risk of coronary heart disease

**DOI:** 10.1186/s13148-023-01464-2

**Published:** 2023-03-28

**Authors:** Jiahui Si, Lu Chen, Canqing Yu, Yu Guo, Dianjianyi Sun, Yuanjie Pang, Iona Y. Millwood, Robin G. Walters, Ling Yang, Yiping Chen, Huaidong Du, Shixian Feng, Xiaoming Yang, Daniel Avery, Junshi Chen, Zhengming Chen, Liming Liang, Liming Li, Jun Lv, the China Kadoorie Biobank Collaborative Group, Junshi Chen, Zhengming Chen, Rory Collins, Liming Li, Richard Peto, Daniel Avery, Ruth Boxall, Derrick Bennett, Yumei Chang, Yiping Chen, Zhengming Chen, Robert Clarke, Huaidong Du, Simon Gilbert, Alex Hacker, Michael Holmes, Andri Iona, Christiana Kartsonaki, Rene Kerosi, Ling Kong, Om Kurmi, Garry Lancaster, Sarah Lewington, Kuang Lin, John McDonnell, Winnie Mei, Iona Millwood, Qunhua Nie, Jayakrishnan Radhakrishnan, Sajjad Rafiq, Paul Ryder, Sam Sansome, Dan Schmidt, Paul Sherliker, Rajani Sohoni, Iain Turnbull, Robin Walters, Jenny Wang, Lin Wang, Ling Yang, Xiaoming Yang, Zheng Bian, Ge Chen, Yu Guo, Can Hou, Jun Lv, Pei Pei, Shuzhen Qu, Yunlong Tan, Canqing Yu, Zengchang Pang, Ruqin Gao, Shaojie Wang, Yongmei Liu, Ranran Du, Yajing Zang, Liang Cheng, Xiaocao Tian, Hua Zhang, Silu Lv, Junzheng Wang, Wei Hou, Jiyuan Yin, Ge Jiang, Xue Zhou, Liqiu Yang, Hui He, Bo Yu, Yanjie Li, Huaiyi Mu, Qinai Xu, Meiling Dou, Jiaojiao Ren, Shanqing Wang, Ximin Hu, Hongmei Wang, Jinyan Chen, Yan Fu, Zhenwang Fu, Xiaohuan Wang, Min Weng, Xiangyang Zheng, Yilei Li, Huimei Li, Yanjun Wang, Ming Wu, Jinyi Zhou, Ran Tao, Jie Yang, Chuanming Ni, Jun Zhang, Yihe Hu, Yan Lu, Liangcai Ma, Aiyu Tang, Shuo Zhang, Jianrong Jin, Jingchao Liu, Zhenzhu Tang, Naying Chen, Ying Huang, Mingqiang Li, Jinhuai Meng, Rong Pan, Qilian Jiang, Weiyuan Zhang, Yun Liu, Liuping Wei, Liyuan Zhou, Ningyu Chen, Hairong Guan, Xianping Wu, Ningmei Zhang, Xiaofang Chen, Xuefeng Tang, Guojin Luo, Jianguo Li, Xiaofang Chen, Xunfu Zhong, Jiaqiu Liu, Qiang Sun, Pengfei Ge, Xiaolan Ren, Caixia Dong, Hui Zhang, Enke Mao, Xiaoping Wang, Tao Wang, Xi zhang, Ding Zhang, Gang Zhou, Shixian Feng, Liang Chang, Lei Fan, Yulian Gao, Tianyou He, Huarong Sun, Pan He, Chen Hu, Qiannan Lv, Xukui Zhang, Min Yu, Ruying Hu, Hao Wang, Yijian Qian, Chunmei Wang, Kaixue Xie, Lingli Chen, Yidan Zhang, Dongxia Pan, Yuelong Huang, Biyun Chen, Li Yin, Donghui Jin, Huilin Liu, Zhongxi Fu, Qiaohua Xu, Xin Xu, Hao Zhang, Youping Xiong, Huajun Long, Xianzhi Li, Libo Zhang, Zhe Qiu

**Affiliations:** 1 Institute of Medical Technology, Health Science Center of Peking University, Beijing, China; 2 National Institute of Health Data Science at Peking University, Peking University, Beijing, China; 3 Department of Epidemiology and Biostatistics, School of Public Health, Peking University Health Science Center, 38 Xueyuan Road, Beijing, 100191 China; 4 Center for Public Health and Epidemic Preparedness & Response, Peking University, 38 Xueyuan Road, Beijing, 100191 China; 5 Fuwai Hospital Chinese Academy of Medical Sciences, Beijing, China; 6 Medical Research Council Population Health Research Unit at the University of Oxford, Oxford, UK; 7 Clinical Trial Service Unit and Epidemiological Studies Unit (CTSU), Nuffield Department of Population Health, University of Oxford, Oxford, UK; 8 NCDs Prevention and Control Department, Henan CDC, Zhengzhou, Henan China; 9 China National Center for Food Safety Risk Assessment, Beijing, China; 10 Departments of Epidemiology and Biostatistics, Harvard T.H. Chan School of Public Health, Boston, MA USA

**Keywords:** Epigenetic age, Cardiovascular health, Coronary artery disease

## Abstract

**Background:**

DNA methylation clocks emerged as a tool to determine biological aging and have been related to mortality and age-related diseases. Little is known about the association of DNA methylation age (DNAm age) with coronary heart disease (CHD), especially in the Asian population.

**Results:**

Methylation level of baseline blood leukocyte DNA was measured by Infinium Methylation EPIC BeadChip for 491 incident CHD cases and 489 controls in the prospective China Kadoorie Biobank. We calculated the methylation age using a prediction model developed among Chinese. The correlation between chronological age and DNAm age was 0.90. DNA methylation age acceleration (Δage) was defined as the residual of regressing DNA methylation age on the chronological age. After adjustment for multiple risk factors of CHD and cell type proportion, compared with participants in the bottom quartile of Δage, the OR (95% CI) for CHD was 1.84 (1.17, 2.89) for participants in the top quartile. One SD increment in Δage was associated with 30% increased risk of CHD (OR = 1.30; 95% CI 1.09, 1.56; Ptrend = 0.003). The average number of cigarette equivalents consumed per day and waist-to-hip ratio were positively associated with Δage; red meat consumption was negatively associated with Δage, characterized by accelerated aging in those who never or rarely consumed red meat (all *P* < 0.05). Further mediation analysis revealed that 10%, 5% and 18% of the CHD risk related to smoking, waist-to-hip ratio and never or rarely red meat consumption was mediated through methylation aging, respectively (all *P* for mediation effect < 0.05).

**Conclusions:**

We first identified the association between DNAm age acceleration and incident CHD in the Asian population, and provided evidence that unfavorable lifestyle-induced epigenetic aging may play an important part in the underlying pathway to CHD.

**Supplementary Information:**

The online version contains supplementary material available at 10.1186/s13148-023-01464-2.

## Background

The risk of coronary heart disease (CHD) strikingly increases with aging [1]. Both genetic and environmental factors are among the determinants of aging [2]. Chronological age, as a most informative marker of aging, cannot depict the remarkable heterogeneity of biological aging rates observed in peers. DNA methylation clocks form an interface between the genotype and the environment [3], emerging as a tool to determine biological aging objectively [4]. Previous studies provided evidence that accelerated DNA methylation age was predictive of mortality [5] and age-related diseases [6] even after adjusting for various known risk factors.

However, little is known about the association of DNA methylation age with CHD. Only a few studies were conducted in European ancestry and got mixed results [7–12]. No previous studies investigated the association of methylation aging with CHD risk in the Asian population, despite the mortality rate of CHD has been increasing continuously in the Asian population [13]. Studies are warranted to increase the genetic diversity of study participants to understand the mechanisms underlying DNA methylation aging across humans [14]. In addition, the current knowledge of how environmental factors affect methylation aging is still lacking [15]. Such factors may serve as intervention targets that might delay cardiovascular aging, thus approaching the goal of longevity.

To fill this gap, we aim to investigate the association between methylation aging and the incident risk of CHD in a Chinese population, and further quantify how much of the effects of lifestyle factors on CHD are mediated through methylation aging. We did a case–control study nested in the 10-year follow-up of the China Kadoorie Biobank (CKB) cohort, comprising 494 incident CHD cases and 494 matched controls.

## Results

The mean chronological age at baseline was 51.1 ± 7.7 years for incident CHD cases and 49.9 ± 7.2 years for matched controls (Table 1). Incident CHD cases were more likely to smoke, have higher measures of adiposity and total cholesterol (TC), and have a higher prevalence of hypertension and diabetes at baseline, compared with matched controls.

**Table 1:** Baseline characteristics of 980 participants according to the case and control status

Baseline characteristics	Cases (*n* = 491)	Controls (*n* = 489)
*Matched factors*		
Age, year	51.1	49.9
Female, %	43.6	43.8
Urban area, %	20.6	20.4
Fasting time, h	4.0	4.0
Middle school and above, %	44.6	44.4
Married, %	90.3	94.1
Family history of heart attack, %	6.9	4.7
*Lifestyle factors*		
Daily tobacco smoker, %	46.5	40.2
Daily alcohol drinker, %	8.9	10.1
Physical activity, MET-h/day	22.3	23.7
*Dietary habits*		
Days consumed fresh vegetables/week	6.8	6.7
Days consumed fresh fruits/week	1.7	2.2
Days consumed red meat/week	2.9	3.0
*Adiposity*		
Body mass index, kg/m^2^	23.9	23.3
Waist circumference, cm	82.2	79.7
Waist-to-hip ratio	0.91	0.88
Fat percentage, %	27.1	25.8
Prevalent hypertension, %	52.0	30.6
Prevalent diabetes, %	9.6	4.7
Total cholesterol, mmol/L	4.7	4.5

The results are presented as means or percentages with adjustment for age, sex, and study area. *MET* Metabolic equivalent of task

### DNAm age and incident CHD

The correlation between chronological age and methylation age was 0.89 and 0.91 in cases and controls, respectively (Additional file 2: Fig. 1). The mean DNAm age was 51.4 ± 6.9 for incident CHD cases and 49.6 ± 6.3 years for matched controls. The corresponding mean value of DNAm age acceleration was 0.42 years for incident cases and − 0.42 years for controls at baseline.

**Fig. 1:**
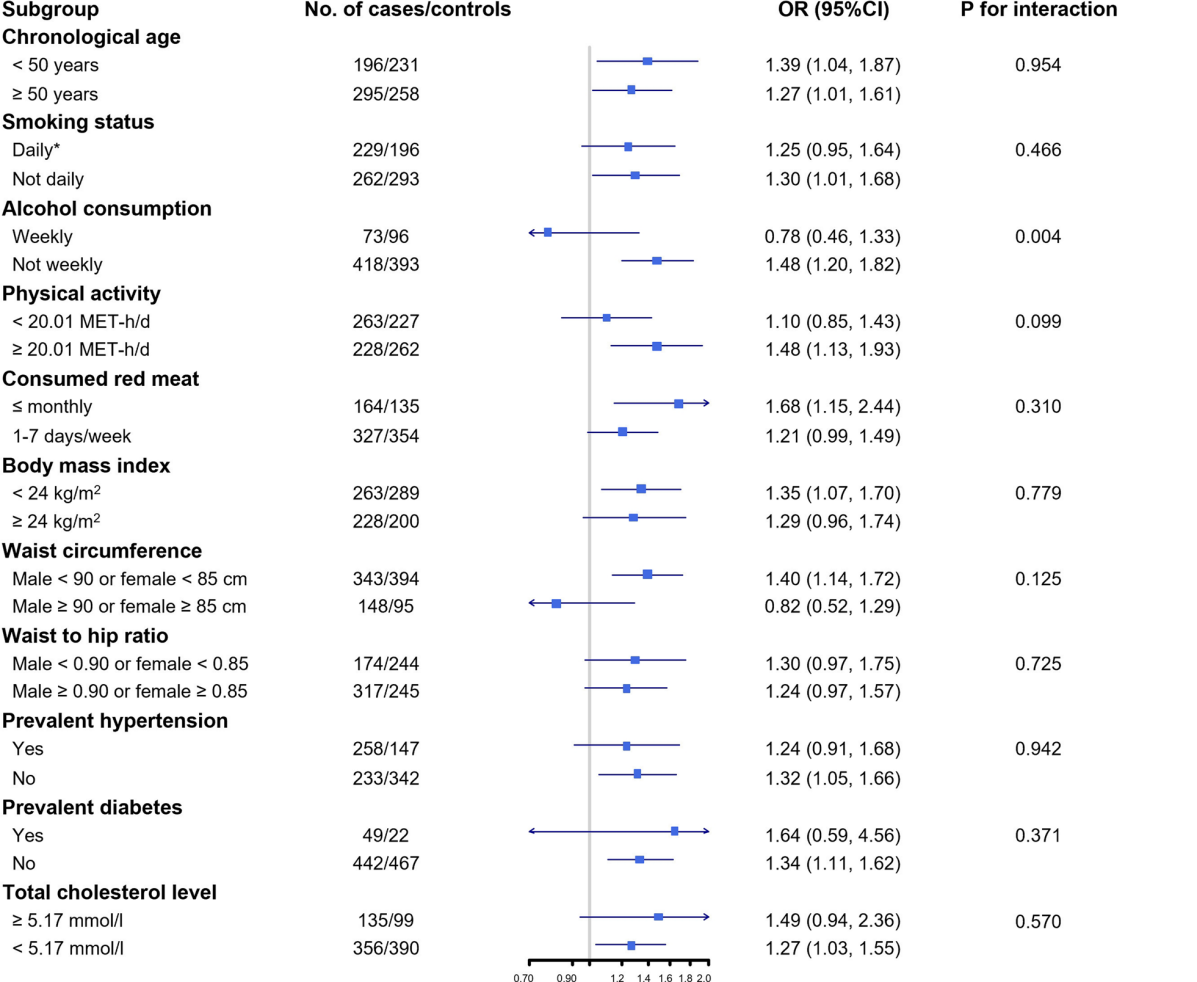
Subgroup analysis of the association between DNA methylation age acceleration and the risk of incident coronary heart disease according to potential baseline risk factors. Horizontal lines represent 95% CIs. Odds ratio and 95% CI are for the associations of 1-SD Δage increasing with CHD risk. Covariates in the multivariable model: age, sex, education level, marital status, smoking, alcohol drinking, physical activity, average days consuming fresh vegetables, fruits, and red meat per week, body mass index, fasting time, study area, batch, and cell type proportions. * To avoid misleadingly elevated risk, former smokers who stopped smoking for illness were categorized as the current smoker

After adjustment for multiple risk factors of CHD and cell type proportion, Δage was associated with increased risk of incident CHD (Table 2). In all eligible participants, compared with participants in the bottom quartile of Δage, the odds ratio (OR) (95% confidence interval [CI]) for CHD was 1.84 (1.17, 2.89) for participants in the top quartile. One SD increment in Δage was associated with 30% increased risk of CHD (OR = 1.30; 95% CI 1.09, 1.56; P_trend_ = 0.003). Further adjustment for baseline cardiometabolic risk factors only slightly attenuated the association (OR = 1.29; 95% CI 1.08, 1.55; P_trend_ = 0.005). The adjusted OR (95%CI) for the risk of CHD associated with one SD increase in Δage was 1.36 (1.07, 1.74) among men and 1.18 (0.89, 1.56) among women. We did not observe statistically significant difference in the association of Δage with CHD between men and women (P_interaction_ = 0.995). We performed conditional logistic regression accounting for matched study design, and additionally adjusted for family history of heart attack. We observed generally similar results in these sensitivity analyses (Additional file 3: Table S1 and S2).

**Table 2:** Odds ratios (95% confidence intervals) for incident coronary heart disease according to DNA methylation age acceleration

	Q1	Q2	Q3	Q4	per SD increase	*P* for trend
Whole cohort						
No. of cases/controls	100/145	118/127	129/116	144/101	491/489	
Multivariable adjusted^*^	1.00	1.37 (0.90, 2.08)	1.72 (1.12, 2.62)	2.02 (1.32, 3.11)	1.36 (1.15, 1.61)	< 0.001
+ cell type proportion†	1.00	1.38 (0.89, 2.14)	1.68 (1.08, 2.62)	1.84 (1.17, 2.89)	1.30 (1.09, 1.56)	0.003
+ baseline cardiometabolic risk factor**	1.00	1.34 (0.85, 2.11)	1.78 (1.13, 2.80)	1.78 (1.12, 2.82)	1.29 (1.08, 1.55)	0.005
*Men*						
No. of cases/controls	62/78	62/78	76/68	77/51	277/275	
Multivariable adjusted^*^	1.00	1.30 (0.73, 2.32)	1.67 (0.95, 2.95)	2.20 (1.22, 3.99)	1.39 (1.10, 1.76)	0.006
+ cell type proportion†	1.00	1.51 (0.81, 2.80)	1.94 (1.06, 3.54)	2.10 (1.12, 3.96)	1.36 (1.07, 1.74)	0.014
+ baseline cardiometabolic risk factor^**^	1.00	1.51 (0.80, 2.85)	2.03 (1.09, 3.75)	1.98 (1.04, 3.77)	1.35 (1.05, 1.74)	0.019
*Women*						
No. of cases/controls	38/67	56/49	53/48	67/50	214/214	
Multivariable adjusted^*^	1.00	1.54 (0.79, 2.98)	1.91 (0.97, 3.76)	1.79 (0.93, 3.46)	1.29 (1.00, 1.67)	0.053
+ cell type proportion†	1.00	1.38 (0.68, 2.79)	1.49 (0.72, 3.10)	1.47 (0.72, 3.00)	1.18 (0.89, 1.56)	0.247
+ baseline cardiometabolic risk factor^**^	1.00	1.40 (0.67, 2.89)	1.66 (0.77, 3.57)	1.51 (0.72, 3.15)	1.17 (0.87, 1.56)	0.301

^*^Covariates in the multivariable model: age, sex, education level, marital status, smoking, alcohol drinking, physical activity, average days consuming fresh vegetables, fruits, and red meat per week, body mass index, fasting time, study area, and batch

^†^The estimated proportion of CD4 + T cell, CD8 + T cell, B cell, natural killer, monocytes, and granulocyte

^**^Baseline cardiometabolic risk factors included prevalent diabetes, hypertension, and total cholesterol level

We examined the association between Δage and CHD risk according to potential baseline risk factors. The positive association between Δage and CHD was stronger in non-weekly drinkers (P_interaction_ = 0.004; Fig. 1). No other statistically significant difference between groups was observed for the following baseline factors: chronological age, smoking status, physical activity, frequency of consuming red meat, body mass index (BMI), waist circumference (WC), waist-to-hip ratio, prevalent hypertension, prevalent diabetes, and TC level.

### Lifestyle factors, DNAm age acceleration, and mediation analysis for CHD

The average number of cigarette equivalents consumed per day was positively associated with Δage (effect size = 0.008; standard error [SE] = 0.003; *P* = 0.017; Table 3). Further mediation analysis revealed that 10.0% of the smoking-associated CHD risk was mediated through Δage (*P* value for average causal mediation effect [ACME] = 0.025). The frequency of consuming red meat was negatively associated with Δage, with the adjusted SD difference (SE) being -0.041 (0.015) per one-day increase in days consuming red meat per week (*P* = 0.007). A total of 17.8% of the red meat-associated CHD risk was mediated through Δage (P_ACME_ = 0.008). The total effect and average direct effect of the average number of cigarette equivalents consumed per day and red meat consumed days per week on CHD were not statistically significant (all *P* value > 0.05; Additional file 3: Table S3). One unit increase in waist-to-hip ratio was associated with a 1.53 SD increase in Δage (SE = 0.566, *P* = 0.007) after adjustment for BMI. The total effect and average direct effect of per 0.1 increase in waist-to-hip ratio were 1.79 (1.56, 1.94) and 1.74 (1.49, 1.90), respectively (all *P* value < 0.001, Additional file 3: Table S3). The proportions of CHD risk associated with waist-to-hip ratio mediated by Δage were 4.7% (P_ACME_ = 0.017). Alcohol consumption, frequency of consuming fresh vegetables and fruits, physical activity, BMI, WC, and fat percentage were not significantly associated with Δage (all *P* values > 0.05; Table 3).

**Table 3:** Associations between lifestyle factors and Δage, and the risk of coronary heart disease mediated through Δage

	Lifestyle factors and Δage *		Mediation effect	
	Effect size (SE)	*P* value	Proportion mediated, %	*P* value for ACME
*Smoking*				
No. of cigarette equivalents/day†	**0.008 (0.003)**	**0.017**	**10.0**	**0.025**
*Alcohol drinking*				
Average pure alcohol (g)/day	0.0004 (0.001)	0.777		
*Dietary habits*				
Fresh vegetables, days/week	0.020 (0.033)	0.551		
Fresh fruits, days/week	0.014 (0.015)	0.354		
Red meat, days/week	**−0.041 (0.015)**	**0.007**	**17.8**	**0.008**
Physical activity (METs-h)	0.002 (0.002)	0.387		
*Adiposity*				
Body mass index (kg/m^2^)	0.012 (0.009)	0.168		
Waist circumference, cm **	0.008 (0.006)	0.186		
Waist-to-hip ratio **	**1.530 (0.566)**	**0.007**	**4.7**	**0.017**
Fat percentage (%)**	−0.008 (0.009)	0.371		

*ACME* Average causal mediation effects; Bold text represents statistically significant results (*P* values < 0.05)

^*^Basic adjustment included age, sex, study area, fasting time, education level, marital status, batch, and the estimated proportion of CD4 + T cell, CD8 + T cell, B cell, natural killer, monocytes, and granulocyte. All lifestyle factors were included in the model simultaneously

^†^ To avoid misleadingly elevated risk, former smokers who stopped smoking for illness were categorized as the current smoker

^**^Additionally adjusted for body mass index (kg/m^2^)

When we included smoking, alcohol consumption, and dietary habits as categorical variables in the analyses of lifestyle factors and Δage, participants who quit smoking because of illness and participants who never or rarely consumed red meat had statistically significantly higher Δage (Additional file 3: Table 4).

## Discussion

In this prospective study of middle-aged Chinese, an understudied population for epigenetics and cardiovascular disparities, we observed epigenetic age acceleration was associated with an increased risk of incident CHD after careful adjustment for potential confounders. One SD increase in DNAm age acceleration was associated with a 30% increased risk of CHD. Further mediation analyses revealed the possible mediating role of DNAm age acceleration in the pathway from smoking, none or rare intake of red meat, and central obesity as measured by waist-to-hip ratio to the risk of CHD.

Individuals of different ethnicities may exhibit different aging rates, methylation profiles, and association effects [7]. Ethnic differences might limit the accuracy of methylation age predictors and also the generalization of the association results across populations. We adopted a methylation aging marker [20] established especially for Chinese populations and observed a strong correlation between methylation aging and chronological age.

Previous studies regarding the association between DNAm age acceleration and CHD were inconsistent. Studies conducted in the European ancestry got mixed results: null [7, 8] or positive association of methylation aging with CHD death [9] and incidence [10]. Only a few studies investigated the impact of methylation aging on CHD among other ancestries [11, 12]. These studies both adopted DNAm GrimAge [11], a composite surrogate biomarker of chronological age, sex, plasma protein levels, and smoking pack-years, and showed increased CHD incidence with accelerated DNAm GrimAge in 1,100 primarily hypertensive African Americans [12] and 6,935 individuals comprising three ethnic groups (50% European ancestry, 40% African Americans, and 10% Hispanic ancestry) [11]. To our knowledge, our study is the first to investigate the association of accelerated epigenetic age with the risk of developing CHD in the Asian population. Our findings expanded the diversity of epigenetic research to understand the mechanisms underlying methylation aging variability across humans.

Lifestyle factors that affect the DNAm markers of aging remain an open question. Studies reported null [23–26] or positive association [8, 27] between smoking behavior and DNAm age among the Western populations; only one study was conducted in African Americans [28]. The majority of previous studies reported null association when comparing the methylation aging of current smokers with others [23, 25, 26, 28]. A previous study showed that smoking pack-year was associated with methylation aging [27]. The present study identified methylation aging accelerated with the average number of cigarette equivalents consumed per day, suggesting that the amount a person smoked is of importance in the methylation aging process. The study of African Americans showed that accelerated methylation aging was observed in participants who quit ≤ 15 years prior, but not in those who quit > 15 years [28]. The present study further identified accelerated methylation aging in former smokers who stopped smoking for illness, but not in those who stopped for other reasons. Taken together, although studies have shown that DNA methylation changes can be reversed following smoking cessation, smoking cessation due to illness or for a short period might be the exception. The differences in methylation aging among former smokers (different cessation duration or reasons) and current smokers (how much has smoked) could be leveraged to offer new insights to explore the effect of smoking on methylation aging in future studies.

BMI was positively associated with DNAm age in previous studies among the Western populations. [8, 25, 27, 29] The present study reported no association of BMI with methylation aging but a positive association of waist-to-hip ratio with methylation aging. Of note, the pattern of obesity is different between Asian and White populations. Asians tend to put on abdominal fat preferentially [30]. Our results indicated that methylation aging might be more sensitive in response to the change in waist-to-hip ratio than BMI in the Asian population. We further quantitatively estimated how much lifestyle factors' effects on CHD are mediated through methylation aging. Our mediation analysis noted that around 10% and 5% of the increased CHD risk related to smoking and central obesity, as measured by waist-to-hip ratio, was mediated through methylation aging, respectively.

Regarding diet, previous studies in the European populations investigated the association between the amount of red meat intake (servings/day) and methylation aging, and found that excess intake of red meat may accelerate the methylation aging rate [25, 31]. Our baseline survey was conducted during 2004–2008. During this time period, the average intake of total meat was 80 g per day in China [32], far less than the 175 g in the U.S [33]. The present study collected data on the frequency of red meat intake (days/week) and found that participants who never or rarely consumed red meat had accelerated methylation aging. These findings might suggest that red meat consumption showed a U-shaped association with methylation aging.

In addition, we found that methylation aging mediated 18% of the CHD risk related to none or rare red meat consumption. The analysis based on 500,000 CKB participants found that lower frequency of red meat intake was associated with a higher risk of intracerebral haemorrhage [34]. Our findings may partially explain the protective impact of moderate red meat consumption on this CVD subtype in Chinese and stimulate future studies toward a better understanding of disease mechanisms.

We firstly investigated the association between DNAm age acceleration and the risk of incident CHD in the Asian population, and quantitatively estimated how much of the effects of unfavorable lifestyles on the increased risk of CHD are mediated through DNAm age acceleration. These findings highlighted the importance of DNA methylation in the underlying mechanisms of cardiovascular disease, and the potential usage of epigenetic age as a biomarker of aging and CHD development. Other strengths included the prospective design that allowed us to preclude the possibility that the changes in DNAm age acceleration were a result of disease state; the use of an accurate methylation age predictor specific for the Chinese population; and careful adjustment of possible risk factors of CHD.

Our study has limitations that warrant discussion. First, the methylation aging was measured in whole blood, which might capture only particular aspects of aging. However, whole blood is easy to access and thus widely used in epidemiological studies. We have made adjustments for cellular compositions, suggesting that our findings were not significantly confounded by the mixed cellular nature of whole blood. These association results may not be generalized to methylation aging of other tissues. Multi-tissue age predictor that fits Chinese population might offer further insights into the aging process. Second, both lifestyle factors and DNA methylation were measured at baseline; therefore, the temporal order between lifestyle and aging acceleration cannot be inferred. However, behavioral lifestyles are long-term habits and less likely to be driven by DNA methylation levels. Changes in covariates during follow-up might lead to residual confounding in prospective studies.

## Conclusions

In this prospective cohort of the Chinese population, we have shown that epigenetic aging was associated with the incident risk of CHD in the next 10 years. Our results also provide evidence that smoking, central obesity, or never consumed red meat-induced epigenetic aging may play an important role in the underlying pathway to CHD. Our study adds to the evidence regarding the potential of using epigenetic clock as a marker of biological age across ethnic diversity humans. Our study also extended beyond the previous evidence that lifestyle intervention may attenuate methylation aging, and further lower the risk of CHD. Future research is warranted to translate epigenetic age acceleration measures into practical clinical and public health applications.

## Methods

### Study population

The CKB cohort was established in 10 geographically diverse areas across China (5 urban and 5 rural sites) during 2004–2008. All participants (*N* = 512,715) had baseline data collected by laptop-based questionnaires, including sociodemographics, lifestyle factors, and medical and medication history. Trained staff measured body weight, height, WC, hip circumference, and body fat percentage using standard protocol and calibrated instruments. All participants provided a 10 ml blood sample for long-term storage, with the time since last meal recorded. Participants were not asked to be fasting. Further details of the CKB have been described elsewhere [16].

The study protocol was approved by the Ethics Review Committee of the Chinese Center for Disease Control and Prevention (005/2004, Beijing, China) and the Oxford Tropical Research Ethics Committee, University of Oxford (025–04, UK). All participants provided written informed consent.

### Study design and DNAm profiling

A total of 494 incident CHD cases occurring before the censoring date of 31 December 2015 and 1:1 matched controls were selected for genome-wide DNA methylation measurements in a case–control study nested in the CKB cohort. Matched factors included age at baseline (± 3 years), birth year (± 3 years), study area, sex, and hours fasting prior to blood draw (0 to < 6, 6 to < 8, 8 to < 10, and ≥ 10 h) at baseline. All these participants were free of heart disease, stroke, or cancer at baseline. Details of the inclusion and exclusion of participants have been provided previously [18].

Incident CHD cases were identified through linkage with the national health insurance system, with local death and disease registries, and by active follow-up. Linkage to the health insurance databases has been achieved 97% of the participants since 2014. Trained staff coded all diagnoses by the International Classification of Diseases, Tenth Revision (ICD-10). Incident CHD included fatal ischemic heart disease (I20-I25 in ICD-10) and nonfatal acute myocardial infarction (I21). Trained staff reviewed hospital medical records for 134 reported cases for diagnosis adjudication, and confirmed 90% of the diagnoses of CHD.

The Infinium Methylation EPIC BeadChip (Illumina, USA), which interrogates ~ 850,000 CpG sites, was used to measure epigenome-wide methylation levels for CHD cases and matched controls (BGI, China). DNA was extracted from baseline peripheral blood leukocytes. *β*-value for each CpG site was reported (ranging from 0 to 1.0) to signify the percentage of DNAm at each CpG site. The R package minfi [17] was used to process methylation data. Quality control, filtering, and normalization of the methylation data have been described previously [18]. These filtering processes resulted in 980 samples with 747,726 CpG sites retained.

### Assessment of lifestyle factors

Lifestyle factors included smoking, alcohol consumption, total physical activity level, dietary habits (fresh vegetables, fruits, and red meat), and adiposity levels (BMI, WC, waist-to-hip ratio, and fat percentage).

Lifestyle factors were collected based on questionnaires and physical measurements. We asked about smoking frequency, type and amount of tobacco smoked per day for ever smoker and calculated the average number of cigarette equivalents consumed per day. We also asked former smokers to report their reason for quitting smoking. Former smokers who stopped smoking for illness were included in the category of current smokers to avoid misleadingly elevated risk. Drinking frequency, type of alcoholic beverage, and volume of alcohol consumed on a typical drinking day were collected at baseline to calculate the average pure alcohol volume consumed per day. The usual type and duration of activities were collected to calculate daily level of physical activity by multiplying the METs value for a particular type of physical activity by hours spent on that activity per day and summing the MET-hours for all activities. Information on consumption frequency of three food items (fresh vegetables, fresh fruits, and red meats) that were mainly addressed in a 2013 guideline from the American Heart Association and the American College of Cardiology on lifestyle management to reduce cardiovascular risk [19] was collected. We then calculated average number of days consuming that food item per week. We calculated BMI (weight in kilograms divided by the square of the height in meters) to measure general adiposity. WC and waist-to-hip ratio (the ratio of WC to hip circumference) were used to measure central adiposity. Body fat percentage was estimated using a TBF-300 monitor (Tanita, Tokyo, Japan).

### Assessment of covariates

For each participant, sociodemographic characteristics (age, sex, education, and marital status) and personal health and medical history (hypertension and diabetes) were also collected based on questionnaire. Trained staff measured blood pressure with calibrated instruments. Participants with measured systolic blood pressure ≥ 140 mm Hg, measured diastolic blood pressure ≥ 90 mm Hg, or self-reporting prior diagnosis of hypertension or usage of antihypertensive medication at baseline were defined as having prevalent hypertension. Participants also provided blood samples for a quick on-site plasma glucose test at baseline. Participants with measured fasting blood glucose ≥ 7.0 mmol/l, measured non-fasting blood glucose ≥ 11.1 mmol/l, or self-reporting prior diagnosis of diabetes were defined as having prevalent diabetes. TC level was measured using standard clinical chemistry assays (Wolfson Laboratory at the University of Oxford, UK).

## Statistical analysis

### DNA methylation age and age acceleration

We calculated the methylation age using an existing prediction model developed among Chinese [20]. This method has been shown to have higher accuracy and less error in Chinese populations [20]. Of all 239 CpGs used to calculate methylation age, 205 available CpGs passed the quality control process. DNA methylation age acceleration (Δage) was defined as the residual of regressing DNA methylation age on the chronological age. Δage was inverse normal transformed (SD = 1) for comparison purposes.

### Δage and incident CHD

Logistic regression was applied to estimate the OR and 95% CI, with adjustment for chronological age (continuous, year), sex (male or female), education level (middle school and above, or others), marital status (married or not), smoking (continuous, average cigarettes or equivalents consumed per day), alcohol consumption (continuous, average pure alcohol volume consumed per day), physical activity (continuous, MET-h/d), average days consuming fresh vegetables, fruits, and red meat per week, BMI (continuous, kg/m [2]), fasting time (0- < 6, 6- < 8, 8- < 10, or ≥ 10 h), ten study area, and five DNAm measurement batchs (Model 1). We additionally adjusted for cellular proportions (CD4 + T cell, CD8 + T cell, B cell, natural killer, monocytes, and granulocyte) (Model 2) and baseline cardiometabolic risk factors (prevalent diabetes, hypertension, and TC level) (Model 3). The proportion of leucocyte cells was estimated from DNAm signature using the algorithm from Houseman et al. [21] by R package minfi [17]. To examine the robustness of the findings, we also performed conditional logistic regression, which accounts for matching, to estimate OR and CI. Matched factors (chronological age, sex, study area, and fasting time) were not included as covariates in the model. We also additionally adjusted for family history of heart attack (yes or no).

We examined the association of Δage with CHD among prespecified baseline subgroups based on sex (men or women), chronological age at baseline (< 50, or ≥ 50 years), smoking status (current daily smoker or not), alcohol consumption (current weekly drinker or not), level of physical activity (categorized using median cutoff), consumption of red meat (1–7 days/week, or ≤ monthly), BMI (< 24.0 or ≥ 24.0 kg/m^2^), WC (male ≥ 90 or female ≥ 85 cm, or others), waist-to-hip ratio (male ≥ 0.90 or female ≥ 0.85, or others), prevalent diabetes (presence or absence), prevalent hypertension (presence or absence), and TC level (≥ 5.17 mmol/l or not). The tests for interaction were performed employing a likelihood ratio test comparing models with and without cross-product terms.

### Lifestyle factors and Δage

Linear regression was applied to investigate the association between lifestyle factors and Δage. All five lifestyle factors were included in the model as continuous variables simultaneously. Covariates adjusted in the model included chronological age, sex, study area, fasting time, marital status, education level, batch, and the estimated proportion of CD4 + T cell, CD8 + T cell, B cell, natural killer, monocytes and granulocyte. In the analysis of central adiposity level or fat distribution with Δage, additional adjustment for BMI was made. Smoking behavior, alcohol consumption, and dietary habits were also treated as categorical variables in the analyses of lifestyle factors and Δage.

### Mediation analyses

In the subsequent mediation analyses, we focused on lifestyle factors associated with Δage (*P* < 0.05). Causal mediation analyses were performed to estimate how much of the lifestyle-associated CHD risk was mediated through Δage. We used the R package mediation [22] to perform parametric regression models. Two models were estimated for each lifestyle factor. The first was regressing Δage (mediator) on the lifestyle factor (exposure) and covariates. The second one was regressing the risk of CHD (outcome) on the lifestyle factor, Δage, and covariates, allowing for exposure-mediator interactions. Covariates included chronological age, sex, study area, fasting time, education level, marital status, batch, blood cell compositions, and four other lifestyle factors. The mediated proportion was calculated as the mediating effect (average causal mediation effect, ACME) of Δage divided by the total effect on log odds scale.

All analyses were performed with Stata version 14.2 (StataCorp) and *R* software version 3.5.2 (*R* Foundation for Statistical Computing). Statistical significance was set at two-tailed *P* < 0.05.

## Supplementary Information

**Additional file 1**. Supplemental Text Members of the China Kadoorie Biobank collaborative group.

**Additional file 2.** Supplemental Figure.

**Additional file 3.** Supplemental Tables 1–4.

## Data Availability

Details of accessing China Kadoorie Biobank data and details of the data release schedule are available from www.ckbiobank.org/site/Data+Access.

## References

[1] Beard JR, Officer A, de Carvalho IA (2016). The World report on ageing and health: a policy framework for healthy ageing. The Lancet.

[2] de Magalhães JP, Wuttke D, Wood SH, Plank M, Vora C (2012). Genome-environment interactions that modulate aging: powerful targets for drug discovery. Pharmacol Rev.

[3] Rosa-Garrido M, Chapski DJ, Vondriska TM (2018). Epigenomes in cardiovascular disease. Circ Res.

[4] Booth LN, Brunet A (2016). The aging epigenome. Mol Cell.

[5] Marioni RE, Shah S, McRae AF (2015). DNA methylation age of blood predicts all-cause mortality in later life. Genome Biol.

[6] Salameh Y, Bejaoui Y, El Hajj N. DNA methylation biomarkers in aging and age-related diseases. Front Genet [Internet] 2020 [cited 2022 May 18];11. 10.3389/fgene.2020.00171 10.3389/fgene.2020.00171 PMC7076122 32211026

[7] Horvath S, Gurven M, Levine ME (2016). An epigenetic clock analysis of race/ethnicity, sex, and coronary heart disease. Genome Biol.

[8] Dugué P-A, Bassett JK, Joo JE (2018). Association of DNA methylation-based biological age with health risk factors and overall and cause-specific mortality. Am J Epidemiol.

[9] Perna L, Zhang Y, Mons U, Holleczek B, Saum K-U, Brenner H (2016). Epigenetic age acceleration predicts cancer, cardiovascular, and all-cause mortality in a German case cohort. Clin Epigenetics.

[10] Lind L, Ingelsson E, Sundström J, Siegbahn A, Lampa E (2018). Methylation-based estimated biological age and cardiovascular disease. Eur J Clin Invest.

[11] Lu AT, Quach A, Wilson JG (2019). DNA methylation GrimAge strongly predicts lifespan and healthspan. Aging.

[12] Ammous F, Zhao W, Ratliff SM (2021). Epigenetic age acceleration is associated with cardiometabolic risk factors and clinical cardiovascular disease risk scores in African Americans. Clin Epigenetics.

[13] GBD 2017 Causes of Death Collaborators (2018). Global, regional, and national age-sex-specific mortality for 282 causes of death in 195 countries and territories, 1980–2017: a systematic analysis for the Global Burden of Disease Study 2017. Lancet Lond Engl.

[14] Breeze CE, Wong JYY, Beck S, Berndt SI, Franceschini N (2022). Diversity in EWAS: current state, challenges, and solutions. Genome Med.

[15] Dhingra R, Nwanaji-Enwerem JC, Samet M, Ward-Caviness CK (2018). DNA methylation age – environmental influences, health impacts, and its role in environmental epidemiology. Curr Environ Health Rep.

[16] Chen Z, Chen J, Collins R (2011). China Kadoorie Biobank of 0.5 million people: survey methods, baseline characteristics and long-term follow-up. Int J Epidemiol.

[17] Aryee MJ, Jaffe AE, Corrada-Bravo H (2014). Minfi: a flexible and comprehensive bioconductor package for the analysis of Infinium DNA methylation microarrays. Bioinforma Oxf Engl.

[18] Si J, Yang S, Sun D (2021). Epigenome-wide analysis of DNA methylation and coronary heart disease: a nested case-control study. Elife.

[19] Eckel RH, Jakicic JM, Ard JD (2014). AHA/ACC guideline on lifestyle management to reduce cardiovascular risk: a report of the american college of cardiology/American heart association task force on practice guidelines. J Am Coll Cardiol.

[20] Li J, Zhu X, Yu K, et al. Exposure to polycyclic aromatic hydrocarbons and accelerated DNA methylation aging. Environ Health Perspect 126(6): 67005. 10.1289/EHP2773 PMC6108582 29906262

[21] Houseman EA, Accomando WP, Koestler DC (2012). DNA methylation arrays as surrogate measures of cell mixture distribution. BMC Bioinformatics.

[22] Tingley D, Yamamoto T, Hirose K, Keele L, Imai K. Mediation : *R* package for causal mediation analysis. J Stat Softw [Internet] 2014 [cited 2022 Mar 9];59(5). Available from: http://www.jstatsoft.org/v59/i05/

[23] Horvath S, Gurven M, Levine ME (2016). An epigenetic clock analysis of race/ethnicity, sex, and coronary heart disease. Genome Biol.

[24] Gao X, Zhang Y, Breitling LP, Brenner H (2016). Relationship of tobacco smoking and smoking-related DNA methylation with epigenetic age acceleration. Oncotarget.

[25] Quach A, Levine ME, Tanaka T (2017). Epigenetic clock analysis of diet, exercise, education, and lifestyle factors. Aging.

[26] Irvin MR, Aslibekyan S, Do A (2018). Metabolic and inflammatory biomarkers are associated with epigenetic aging acceleration estimates in the GOLDN study. Clin Epigenetics.

[27] McCartney DL, Stevenson AJ, Walker RM (2018). Investigating the relationship between DNA methylation age acceleration and risk factors for Alzheimer’s disease. Alzheimers Dement Amst Neth.

[28] Simons RL, Lei MK, Beach SRH (2016). Economic hardship and biological weathering: the epigenetics of aging in a US sample of black women. Soc Sci Med.

[29] Nevalainen T, Kananen L, Marttila S (2017). Obesity accelerates epigenetic aging in middle-aged but not in elderly individuals. Clin Epigenetics.

[30] Ramachandran A, Chamukuttan S, Shetty SA, Arun N, Susairaj P (2012). Obesity in Asia–is it different from rest of the world. Diabetes Metab Res Rev.

[31] Levine ME, Lu AT, Quach A (2018). An epigenetic biomarker of aging for lifespan and healthspan. Aging.

[32] He Y, Li Y, Yang X (2019). The dietary transition and its association with cardiometabolic mortality among Chinese adults, 1982–2012: a cross-sectional population-based study. Lancet Diabetes Endocrinol.

[33] FoodReview. commodity economics division, economic research service, U.S. Department of agriculture; 2002.

[34] Kakkoura MG, Du H, Key TJ, Chen Z, China Kadoorie Biobank Collaborative Group Associations of red meat, poultry, fish and egg intake with risk of cardiovascular disease: an 11-year prospective study of the China Kadoorie Biobank. Eur Heart J 2021;42(Supplement_1): 724.2438.

