# Supplementary material for: Healthy lifestyle, DNA methylation age acceleration, and incident risk of coronary heart disease
Source: Clin Epigenetics. 2023 Mar 28;15:52. doi: 10.1186/s13148-023-01464-2 (PMC10045869; doi:10.1186/s13148-023-01464-2)
Supplement: Supplementary file 1 — Additional file 1. Supplemental Text Members of the China Kadoorie Biobank collaborative group. [file 13148_2023_1464_MOESM1_ESM.docx]

**Supplemental Text Members of the China Kadoorie Biobank collaborative group:**

International Steering Committee: Junshi Chen, Zhengming Chen (PI), Rory Collins, Liming Li (PI), Richard Peto.

International Co-ordinating Centre, Oxford: Daniel Avery, Ruth Boxall, Derrick Bennett, Yumei Chang, Yiping Chen, Zhengming Chen, Robert Clarke, Huaidong Du, Simon Gilbert, Alex Hacker, Michael Holmes, Andri Iona, Christiana Kartsonaki; Rene Kerosi, Ling Kong, Om Kurmi, Garry Lancaster, Sarah Lewington, Kuang Lin, John McDonnell, Winnie Mei, Iona Millwood, Qunhua Nie, Jayakrishnan Radhakrishnan, Sajjad Rafiq, Paul Ryder, Sam Sansome, Dan Schmidt, Paul Sherliker, Rajani Sohoni, Iain Turnbull, Robin Walters, Jenny Wang, Lin Wang, Ling Yang, Xiaoming Yang. National Co-ordinating Centre, Beijing: Zheng Bian, Ge Chen, Yu Guo, Can Hou, Jun Lv, Pei Pei, Shuzhen Qu, Yunlong Tan, Canqing Yu.

10 Regional Co-ordinating Centres: Qingdao CDC: Zengchang Pang, Ruqin Gao, Shaojie Wang, Yongmei Liu, Ranran Du, Yajing Zang, Liang Cheng, Xiaocao Tian, Hua Zhang. Licang CDC: Silu Lv, Junzheng Wang, Wei Hou. Heilongjiang Provincial CDC: Jiyuan Yin, Ge Jiang, Xue Zhou. Nangang CDC: Liqiu Yang, Hui He, Bo Yu, Yanjie Li, Huaiyi Mu, Qinai Xu, Meiling Dou, Jiaojiao Ren. Hainan Provincial CDC: Shanqing Wang, Ximin Hu, Hongmei Wang, Jinyan Chen, Yan Fu, Zhenwang Fu, Xiaohuan Wang. Meilan CDC: Min Weng, Xiangyang Zheng, Yilei Li, Huimei Li,Yanjun Wang. Jiangsu Provincial CDC: Ming Wu, Jinyi Zhou, Ran Tao, Jie Yang. Suzhou CDC: Chuanming Ni, Jun Zhang, Yihe Hu, Yan Lu, , Liangcai Ma, Aiyu Tang, Shuo Zhang, Jianrong Jin, Jingchao Liu. Guangxi Provincial CDC: Zhenzhu Tang, Naying Chen, Ying Huang. Liuzhou CDC: Mingqiang Li, Jinhuai Meng, Rong Pan, Qilian Jiang, Weiyuan Zhang, Yun Liu, Liuping Wei, Liyuan Zhou, Ningyu Chen, Hairong Guan. Sichuan Provincial CDC: Xianping Wu, Ningmei Zhang, Xiaofang Chen, Xuefeng Tang. Pengzhou CDC: Guojin Luo, Jianguo Li, Xiaofang Chen, Xunfu Zhong, Jiaqiu Liu, Qiang Sun. Gansu Provincial CDC: Pengfei Ge, Xiaolan Ren, Caixia Dong. Maiji CDC: Hui Zhang, Enke Mao, Xiaoping Wang, Tao Wang, Xi zhang. Henan Provincial CDC: Ding Zhang, Gang Zhou, Shixian Feng, Liang Chang, Lei Fan. Huixian CDC: Yulian Gao, Tianyou He, Huarong Sun, Pan He, Chen Hu, Qiannan Lv, Xukui Zhang. Zhejiang Provincial CDC: Min Yu, Ruying Hu, Hao Wang. Tongxiang CDC: Yijian Qian, Chunmei Wang, Kaixue Xie, Lingli Chen, Yidan Zhang, Dongxia Pan. Hunan Provincial CDC: Yuelong Huang, Biyun Chen, Li Yin, Donghui Jin, Huilin Liu, Zhongxi Fu, Qiaohua Xu. Liuyang CDC: Xin Xu, Hao Zhang, Youping Xiong, Huajun Long, Xianzhi Li, Libo Zhang, Zhe Qiu.
