# Supplementary material for: Healthy lifestyle, DNA methylation age acceleration, and incident risk of coronary heart disease
Source: Clin Epigenetics. 2023 Mar 28;15:52. doi: 10.1186/s13148-023-01464-2 (PMC10045869; doi:10.1186/s13148-023-01464-2)

# Supplemental Figure 1 Correlation between DNA methylation age and chronological age among cases of coronary heart disease (CHD) and matched controls.


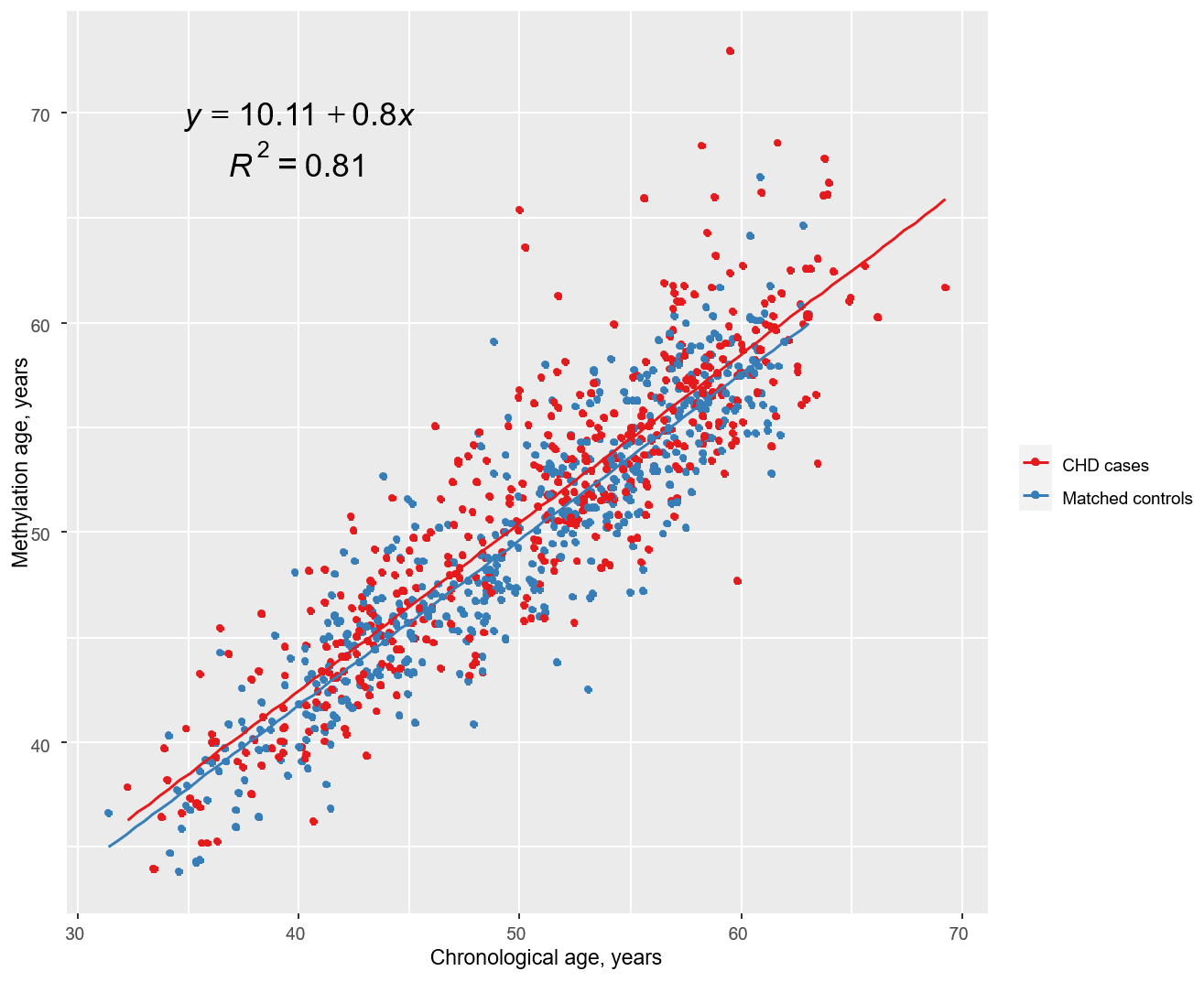

Supplement: Supplementary file 2 — Additional file 2. Supplemental Figure. [file 13148_2023_1464_MOESM2_ESM.docx]
