# Supplementary material for: Healthy lifestyle, DNA methylation age acceleration, and incident risk of coronary heart disease
Source: Clin Epigenetics. 2023 Mar 28;15:52. doi: 10.1186/s13148-023-01464-2 (PMC10045869; doi:10.1186/s13148-023-01464-2)
Supplement: Supplementary file 3 — Additional file 3. Supplemental Tables 1–4. [file 13148_2023_1464_MOESM3_ESM.docx]

# Supplemental Table 1 Odds ratios (95% confidence intervals) for incident coronary heart disease according to DNA methylation age acceleration using logistic or conditional logistic regression.

|  | per SD increase  (logistic regression) | per SD increase  (conditional logistic) |
| --- | --- | --- |
| Whole cohort |  |  |
| Multivariable adjusted^*^ | 1.36 (1.15, 1.61) | 1.36 (1.12, 1.66) |
| +cell type proportion† | 1.30 (1.09, 1.56) | 1.28 (1.04, 1.59) |
| +baseline cardiometabolic risk factor** | 1.29 (1.08, 1.55) | 1.29 (1.03, 1.60) |

*Multivariable model was adjusted for: age, sex, education level, marital status, smoking, alcohol drinking, physical activity, dietary habits, body mass index, fasting time, study area, and batch. Matching factors (age, sex, fasting time, and study area) were excluded when using conditional logistic regression.

†The estimated proportion of CD4+ T cell, CD8+ T cell, B cell, natural killer, monocytes, and granulocyte.

**Baseline cardiometabolic risk factors included prevalent diabetes, prevalent hypertension, and total cholesterol level.

# Supplemental Table 2 Odds ratios (95% confidence intervals) for incident coronary heart disease according to DNA methylation age acceleration with adjustment for family history of heart attack.

|  | Q1 | Q2 | Q3 | Q4 | per SD increase | P for trend |
| --- | --- | --- | --- | --- | --- | --- |
| No. of cases/controls | 100/145 | 118/127 | 129/116 | 144/101 | 491/489 |  |
| Multivariable adjusted^*^ | 1.00 | 1.36 (0.89, 2.07) | 1.70 (1.11, 2.60) | 2.01 (1.31, 3.09) | 1.35 (1.14, 1.60) | <0.001 |
| +cell type proportion† | 1.00 | 1.37 (0.88, 2.13) | 1.65 (1.06, 2.58) | 1.82 (1.15, 2.86) | 1.30 (1.09, 1.55) | 0.004 |
| +baseline cardiometabolic risk factor** | 1.00 | 1.33 (0.85, 2.10) | 1.76 (1.12, 2.77) | 1.76 (1.11, 2.79) | 1.29 (1.08, 1.54) | 0.006 |

*Covariates in the multivariable model: age, sex, education level, marital status, smoking, alcohol drinking, physical activity, average days consuming fresh vegetables, fruits, and red meat per week, body mass index, fasting time, study area, family history of heart attack and batch.

†The estimated proportion of CD4+ T cell, CD8+ T cell, B cell, natural killer, monocytes, and granulocyte.

**Baseline cardiometabolic risk factors included prevalent diabetes, hypertension, and total cholesterol level.

# Supplemental Table 3 Total, direct and mediation effect through Δage of lifestyle factors on the risk of coronary heart disease.

|  |  | Total effect | | Direct effect | | Mediation effect | |
| --- | --- | --- | --- | --- | --- | --- | --- |
|  |  | OR (95%CI) | P value | OR (SE) | P value | Proportion mediated, % | P value for ACME |
|  |  |  |  |  |  |  |  |
| Smoking |  |  |  |  |  |  |  |
| No. of cigarette equivalents/day† |  | 1.003  (0.985, 1.019) | 0.888 | 0.999  (0.983, 1.017) | 0.939 | 10.0 | 0.025 |
| Dietary habits |  |  |  |  |  |  |  |
| Red meat, days/week |  | 0.966  (0.891, 1.047) | 0.400 | 0.977  (0.901, 1.058) | 0.563 | 17.8 | 0.008 |
| Adiposity |  |  |  |  |  |  |  |
| 0.1 increase in waist-to-hip ratio ** |  | 1.793  (1.559, 1.935) | <0.001 | 1.737  (1.488, 1.896) | <0.001 | 4.7 | 0.017 |

*Basic adjustment included age, sex, study area, fasting time, education level, marital status, batch, and the estimated proportion of CD4+ T cell, CD8+ T cell, B cell, natural killer, monocytes, and granulocyte. All lifestyle factors were included in the model simultaneously.

† To avoid misleadingly elevated risk, former smokers who stopped smoking for illness were categorized as the current smoker.

**Additionally adjusted for body mass index (kg/m^2^).

ACME = average causal mediation effects.

# Supplemental Table 4 Associations of smoking, alcohol consumption, and dietary habits with Δage.

|  |  | No. of cases/controls | Lifestyle factors and Δage * | |  |
| --- | --- | --- | --- | --- | --- |
|  |  |  | Effect size (SE) | P value |  |
|  |  |  |  |  |  |
| Smoking behavior |  |  |  |  |  |
| Never smoker |  | 251/278 | Reference | Reference |  |
| Quit because of illness |  | 13/14 | **0.447 (0.196)** | **0.023** |  |
| Quit because of other reasons |  | 11/15 | -0.214 (0.199) | 0.282 |  |
| Current with < 15 cig/day |  | 86/77 | 0.076 (0.107) | 0.475 |  |
| Current with 15 - < 25 cig/day |  | 91/69 | 0.063 (0.110) | 0.568 |  |
| Current with ≥ 25 cig/day |  | 39/36 | 0.200 (0.139) | 0.149 |  |
| Alcohol drinking behavior |  |  |  |  |  |
| Never drinker |  | 396/371 | Reference | Reference |  |
| Former drinker |  | 22/22 | 0.005 (0.150) | 0.973 |  |
| Weekly |  | 29/48 | -0.167 (0.117) | 0.155 |  |
| Daily with < 30 g/day |  | 7/10 | -0.166 (0.231) | 0.473 |  |
| Daily with 30 - 59 g/day |  | 15/18 | -0.076 (0.170) | 0.655 |  |
| Daily with ≥ 60 g/day |  | 22/20 | 0.044 (0.157) | 0.779 |  |
| Dietary habits |  |  |  |  |  |
| Fresh vegetables |  |  |  |  |  |
| Never/rarely |  | 1/1 | 0.851 (0.648) | 0.190 |  |
| Monthly |  | 0/1 | -0.005 (0.913) | 0.995 |  |
| 1-6 days/week |  | 35/42 | -0.033 (0.120) | 0.780 |  |
| Daily |  | 455/445 | Reference | Reference |  |
| Fresh fruits, days/week |  |  |  |  |  |
| Never/rarely |  | 49/43 | 0.083 (0.138) | 0.545 |  |
| Monthly |  | 224/195 | -0.088 (0.108) | 0.413 |  |
| 1-6 days/week |  | 172/183 | -0.064 (0.105) | 0.546 |  |
| Daily |  | 46/68 | Reference | Reference |  |
| Red meat, days/week |  |  |  |  |  |
| Never/rarely |  | 55/43 | **0.226 (0.110)** | **0.041** |  |
| Monthly |  | 109/92 | -0.078 (0.086) | 0.362 |  |
| 1-6 days/week |  | 225/257 | Reference | Reference |  |
| Daily |  | 102/97 | **-0.193 (0.088)** | **0.029** |  |

*Basic adjustment included age, sex, study area, fasting time, education level, marital status, physical activity, body mass index, batch, and the estimated proportion of CD4+ T cell, CD8+ T cell, B cell, natural killer, monocytes and granulocyte. Smoking, alcohol consumption, and dietary habits were included in the model simultaneously.
